# Large-scale evaluation efforts and their implications for the field

**DOI:** 10.1002/ev.20503

**Published:** 2022-08-08

**Authors:** Tarek Azzam

**Affiliations:** University of California, Santa Barbara, California, USA

## Abstract

The BUILD initiative is part of the Diversity Program Consortium, which the National Institutes of Health funded to increase diversity in biomedical research. This chapter aims to identify implications for the field from the multisite evaluation of BUILD initiative programs by reviewing the work undertaken by the authors of the other chapters in this issue. Given the complexities involved in multisite evaluations, innovative approaches and methods were used to balance the needs of each site with the overall objectives of the broader initiative. These approaches included a flexible orientation to the evaluation, mixed-methods designs that prioritized understanding the context before measuring it, and innovative analytic techniques (e.g., meta-analysis) to recognize the uniqueness of each site while providing insights about their cumulative impact. The BUILD initiative evaluation also offered many other valuable lessons about engaging stakeholders, focusing on use, and responding to changing priorities over time.

## INTRODUCTION

Large scale multisite evaluations are difficult yet worthwhile because they force us to consider local contextual factors while maintaining a broader perspective on the overall initiative. This challenge, if approached effectively, has many potential benefits, including a better understanding of how policies can change trends and how they are translated to respond to the needs of different communities. Previous multisite evaluations have tended to focus on differing elements, including the methodological approaches involved in the process (Stachowiak, Lynn, & Akey, 2020), the role of culture and collaboration (Cook, Carey, Razzano, Burke, & Blyler, 2002), and the ability to identify broader outcomes (Straw & Herrell, 2002).

The contribution of this *New Directions for Evaluation* issue to this growing knowledge base is evident. The preceding chapters have explored the complex evaluation of the Building Infrastructure Leading to Diversity (BUILD) initiative, funded by the National Institutes of Health as part of the Diversity Program Consortium (DPC) to increase the diversity of students who pursue biomedical research careers. The evaluation is overseen by the initiative’s Coordination and Evaluation Center (CEC), housed at the University of California, Los Angeles.

As I reviewed the work of my colleagues, I was struck by the myriad methodological considerations, analytic techniques, and responsiveness to context and stakeholders that emerged across this evaluation endeavor. Together, these chapters provide one of the few examples of large-scale multisite evaluations that have been documented and presented from varying perspectives, including the technical, cultural, and theoretical; they also provide new insights that can inform our practice. The hope is that the lessons learned from this project will inform future evaluation efforts across sectors and contexts.

To help frame the insights gained in this chapter I utilize Lee Cronbach’s ideas on generalizability. In his approach to evaluation, Cronbach (1982) argued for the use of multiple studies (often described as a fleet of studies) to understand the impact of programs and policies across various contexts. Rather than investing in a single study that focuses on casual relationships under very controlled environments (e.g., through the use of randomized control trials), Cronbach recommended tracking various elements of an evaluation context, including the people involved, the intervention they received, the measures or observations used to understand the outcomes, and the setting where the intervention took place. This description is often referred to as *utos*, where *u =* units (people); *t =* treatment (intervention); *o =* observation (measures); and *s =* setting (context of intervention).^1^

At its core, this approach attempts to represent how an initiative has responded to the uniqueness of its environment by comparing and contrasting various small evaluation studies of the program. This process can lead to insights about the environmental factors that have enhanced or hindered success within differing contexts. It is also worth noting that Cook (2004) included *time* as fifth element to show how multiyear programs change and evolve as they develop. Overall, this way of representing the context may help offer insights into some of the broad lessons learned from the efforts of the DPC. My goal is to describe its implications to the evaluation field.

## UNDERSTANDING BUILD THROUGH A *UTOS* LENS

The overall purpose of BUILD was described in Chapter 1. Specifically, the initiative has the ultimate goal of “[enhancing] the diversity of well-trained biomedical research scientists who can successfully compete for NIH research funding and/or otherwise contribute to the NIH-funded workforce”^2^. Understanding whether this broad goal has been reached requires knowledge of who was served as part of the program and the types of training needed to support their potential for success.

### BUILD units

The diversity of students and faculty across sites were the *u* in the *utos* model, as these scholars focused on the individuals the program recruited, retained, and supported. From an evaluation perspective, the efforts described in Chapter 5 by Maccalla et al. represented a potentially effective way of understanding who the participants were in terms of their position (student or faculty), relevant information about their role in the program (e.g., Scholar, Associate), and a host of other background characteristics. From an innovation standpoint, the systematic approach described in Chapter 5 allowed the evaluation to reveal how unique individual characteristics across various sites interacted with the contexts within which they were working, while also providing a broader perspective on the achieved outcomes. This ability to view the forest and trees offered information on who participated and how long they remained part of the program and ultimately helped identify interactions between participants and the outcomes that emerged from their program engagement. This also helped answer questions about whom the program worked for and possibly about why it worked for them. Part of that answer also relied on how the “program” was defined at each of the sites.

### BUILD treatments and observations

How the program was defined at each site represents the treatment or *t* in the *utos* framework. As acknowledged in Chapter 1 (and in other chapters), this initiative was implemented heterogeneously across 10 sites, and the programing changed over time. As such, there were differences between sites and within sites over time. The difficulty of disentangling the changing program characteristics across sites and time and then identifying the program’s relationship to various measured outcomes would pose a great challenge to an evaluator. These measures can be the observations, or the *o* in the *utos* framework. Given this challenge, I believe that this evaluation did an excellent job of developing various qualitative and quantitative approaches to capture programmatic activities and outcomes and speak to the potential impacts of these efforts within and across sites.

For example, if we think about the various evaluation activities that the CEC conducted, we can detect a pattern that mimics an exploratory sequential mixed-methods design (Creswell & Clark, 2017). In this design, qualitative information is collected and used to explore the context. Findings from this qualitative phase inform a quantitative phase that can examine the generalizability of the qualitative findings and may also be used to inform the development of quantitative outcome measures. This methodological approach can help evaluators understand how a program is being implemented across various sites (i.e., the treatment or *t* in *utos*), or help the evaluation gain a more accurate understanding of the program outcomes and how to optimally measure them (i.e., the observation or *o* in *utos*).

After reviewing and reflecting on the various chapters as a whole, the mixed-methods approach appeared most likely occurred during the case study portion of the evaluation (see Chapter 2 by Cobian et al.) and when identifying Hallmarks of Success (see Chapter 5 by Maccalla et al.). In the case study portion, the authors noted that “Program evaluation efforts that utilize case study design can provide a rich, in-depth understanding of program effectiveness that can either supplement quantitative evaluation measures in an explanatory, mixed methods evaluation to increase understanding of program outcomes, or stand alone in presenting multi-faceted dimensions of program implementation” (Cobian et al., this issue). This initial qualitative study looked at factors that may have influenced how each site designed an initiative that best suited its specific needs, history, resources, capacity, students, and faculty. This qualitative study also offered insights into *why* there was heterogeneous implementation, and *how* these varying program models connected to the outcomes. I am unsure how the information gathered from the case study informed other pieces of this evaluation, as this was not explicitly stated in the other chapters. Given the structure of the remaining evaluation efforts, the findings from these case studies may have formally or informally guided some of the subsequent quantitative processes present throughout the evaluation.

The exploratory mixed-methods approach was also described by Maccalla et al. in Chapter 5, where an initial qualitative process was implemented to define what a “program” is. This initial qualitative exploratory phase included document review, member checking, and thematic coding to understand the various program components and how they were operationalized across sites. This process also offered insights about the treatment, or the *t* in *utos*. Even though this phase was iterative in nature, it aided the development of a quantitative tracking tool that operationalized many of the qualitatively described program components that emerged from the initial exploratory phase. Quantitative measures included enrollment in new courses developed across the sites and participation in career advancement activities, such as career advising, learning communities, and undergraduate research experiences. The quantitative tracking also extended to faculty involvement in program activities, including research training and support and conference participation.

This tracking system allowed the evaluation to compare program implementation by creating categories of activities that were broad enough to be applicable across the 10 sites yet detailed enough to reflect the unique focus of each site. This approach helped provide the evaluation with a perspective that balanced the forest and trees. Even though the mixed-methods approach was not explicitly stated in the chapter, the approach as described fits the definition of an exploratory sequential mixed-methods design (Creswell & Clark, 2017), with the caveat that it was iterative in nature.

Other approaches were also used to balance contextual factors with the broader implications of the program. Crespi and Cobian clearly illustrated this in Chapter 4, where a primarily quantitative approach was used to capture program outcomes (i.e., observations, or the *o* in *utos*). This has some very interesting implications for the field of evaluation, since the approach leveraged meta-analysis to provide an understanding of what occurred at each site and what happened across sites. As the authors noted, meta-analysis has historically been used to aggregate findings from hundreds or thousands of studies. From a conceptual perspective, a meta-analytic approach takes differing outcome measures from across studies and standardizes the outcomes by calculating their effect sizes, and then uses that information to calculate the absolute effect size to determine the overall impact of an intervention (Borenstein, Hedges, Higgins, & Rothstein, 2021; Lipsey & Wilson, 2001). The effect size measures the strength between two variables or the magnitude of the change that has occurred (Lipsey & Wilson, 2001). The larger the effect size, the larger the relationship between variables or the larger the change over time.

Typically, a researcher conducting a meta-analysis would collect many studies that examine the outcome of interest, even in the gray literature (Lipsey & Wilson, 2001). For example, ‘ Hattie (2013) used meta-analysis to study the impacts of various educational interventions, such as writing programs, inquiry-based teaching, and microteaching, on academic outcomes (e.g., standardized test scores). If the meta-analysis focused on writing programs, then studies that examined different types of writing programs and that utilized various measures of writing would be collected. The program components would then be categorized in terms of activities, while program outcomes would be standardized using the effect-size calculation. This can occur regardless of the methodological design of the study (which, in this case, included randomized control trials, quasi-experimental designs, and pre-experimental designs). The meta-analysis would take these various effects sizes and available contextual variables (e.g., number of participants, type of intervention, type of methodological design) and produce an overall absolute effect size for writing programs.

In evaluation, this absolute effect size could be used to evaluate programs when no comparison groups are available. For example, if you are evaluating a program that is focused on improving student academic performance using microteaching, you can look up the effect size that is typically associated with this type of intervention (effect size = .88; Hattie & Yates, 2013) and compare it to the effect size produced by the program you are evaluating. If your program’s effect size is the same or larger than .88, it provides supporting evidence of its effectiveness. If your program’s effect size is much smaller than .88, this knowledge can serve as evidence that the program may need to be revised to improve its effects. Although this example may be viewed as a digression from the main points of this chapter, I include it to illustrate how meta-analysis and effect sizes can be used in evaluation and to contrast this approach with the innovation described by Crespi and Cobian in Chapter 4.

In Chapter 4, Crespi and Cobian (this issue) used the meta-analytic approach in an innovative way by leveraging its main advantage—the ability to standardize findings from across 10 program sites—and accounting for different sample characteristics and different implementation plans. They calculated these effect sizes for each program site and then used them in the broader meta-analysis to provide an aggregated absolute effect of the entire initiative. This is a relevant contribution to evaluation practice because it permits different sites to use their own conceptualizations of quantitative outcomes and activities. These outcomes and activities may best represent the unique goals they wish to influence and the activities that would optimally serve their students while still allowing the evaluation to combine findings at a broader level to determine overall initiative impact. This effort also builds on previous work conducted by Banks, McHugo, Williams, Drake, and Shinn (2002), who used similar techniques to evaluate outcomes across multiple program interventions. I hope to see this technique more widely adopted in future multisite evaluations.

### BUILD settings

The last element in the *utos* framework is examining the setting, or the *s*, and the influence of contextual characteristics, such as culture, history, and the surrounding environment, on programs and their outcomes. This element was not described in great detail within the preceding chapters but was captured in the overall evaluation theories that helped guide this effort. As mentioned in Chapter 6 by Christie and Wright, the primary theoretical framework that was adopted was utilization—specifically, utilization-focused evaluation (Patton, 2012) and participatory evaluation (Cousins, & Earl, 1995). There were also elements of culturally responsive evaluation (Hood, Hopson, & Kirkhart, 2015) embedded across the 10 different sites. This theory prioritizes social justice, equity, and advocacy as part of the evaluation effort.

These evaluation theories have a common focus at their core: the importance of understanding and responding to programs and stakeholders. These theories can be flexible rather than impose a rigid and unresponsive evaluation. This is a potentially important advantage, as they may offer a more realistic perspective on how programs adapt to the settings they are placed in and provide useful information in a responsive and culturally respectful manner. Other evaluation theories can be equally flexible in different settings, including responsive evaluations (Stake, 2003) and deliberative democratic evaluations (House & Howe, 2003), and are potentially viable for other multisite evaluations.

In terms of the implications for the evaluation field, multisite programs may be best served through these evaluation theories because they prioritize the interaction between programs and their settings throughout the evaluative process. For example, a utilization-focused evaluation would engage the primary intended users at each site to understand their differing data needs, timelines, strengths, and challenges (Patton, 2012). This site-specific knowledge would be used to design an evaluation that is responsive to these needs and offer data (qualitative, quantitative, or mixed) with the highest potential to be used in the decision-making process. One site may need implementation data on a new program component being piloted; another site may need short-term outcomes to help guide revisions to the intervention.

The evaluation would respond to these needs within a utilization-focused approach across the different sites. The focus would be on providing information that can be used. Other contextually responsive evaluation theories would potentially focus on slightly different elements. For example, a culturally responsive evaluation prioritizes the cultural norms of the community and the participants being served by the program, there would also be an emphasis on social justice as part of the process. Although use may occur due to the responsive nature of the CRE approach, the focus would be on conducting an evaluation that explicitly acknowledges the uniqueness of the setting, the cultural background of individuals, and the power dynamics. Deliberative democratic evaluation would also be similar in its responsiveness to the setting, and its focus would be on inclusion, dialogue, deliberation and social justice. The key advantage of any of the mentioned evaluation theories is their ability to examine, acknowledge, and respond to the unique settings of each site within multisite evaluation.

## CONCLUDING THOUGHTS

Multisite evaluations are challenging due to the variability of the individuals involved, differences in the how the program is implemented, variations in how outcomes are operationalized, and the uniqueness of the environmental context for each site. This issue offers insights into how to approach this challenge. Mixed-methods approaches can help identify variations in implementation across sites and aid in developing outcomes that better reflect each site’s priorities. For example, if an evaluation is working with a new multisite program, the recommendation would be to use an exploratory, sequential, mixed-methods design to understand the context qualitatively before developing quantitative measures. In contrast, if there is an established quantitative outcome that is used at every site, then an explanatory, sequential, mixed-methods design would be a better fit (Creswell & Clark, 2017). This design would help reveal the reasons for any variations in quantitative data across sites.

Evaluation theories that offer flexibility may also be optimally suited for multisite evaluation. They can take into account the characteristics of each site while still using technical methods (such as meta-analysis) to gain a broader perspective on the initiative’s impact. This balancing act between standardization and flexibility is at the core of any multisite evaluation, and it was a tangible struggle in this specific effort. However, I believe that the chapter authors overcame many of these challenges and were able to respond to the uniqueness of each site by thoughtfully acknowledging the individuals, the activities, the outcomes, and the settings while also identifying the overall impacts of this large-scale effort. Many lessons have been learned from this process, and I hope that the evaluation community has gained a better perspective from this valuable work.

## ENDNOTES

1 For a fuller description of this approach please see Greene (2004).

2 Source: https://grants.nih.gov/grants/guide/rfa-files/RFA-RM-13-015.html
